# ATF3 negatively regulates cellular antiviral signaling and autophagy in the absence of type I interferons

**DOI:** 10.1038/s41598-017-08584-9

**Published:** 2017-08-18

**Authors:** Vikas Sood, Kiran Bala Sharma, Vishal Gupta, Dhurjhoti Saha, Parashar Dhapola, Manish Sharma, Utsav Sen, Shigetaka Kitajima, Shantanu Chowdhury, Manjula Kalia, Sudhanshu Vrati

**Affiliations:** 1 Vaccine and Infectious Disease Research Centre, Translational Health Science and Technology Institute, Faridabad, 121001 India; 2 CSIR-Institute of Genomics and Integrative Biology, Delhi, 110007 India; 3 Tokyo Medical and Dental University, Tokyo, 113-0034 Japan; 4 Faculty of Biological Sciences, Academy of Scientific and Innovative Research (AcSIR), New Delhi, 110025 India; 5 Regional Centre for Biotechnology, Faridabad, 121001 India

## Abstract

Stringent regulation of antiviral signaling and cellular autophagy is critical for the host response to virus infection. However, little is known how these cellular processes are regulated in the absence of type I interferon signaling. Here, we show that ATF3 is induced following Japanese encephalitis virus (JEV) infection, and regulates cellular antiviral and autophagy pathways in the absence of type I interferons in mouse neuronal cells. We have identified new targets of ATF3 and show that it binds to the promoter regions of *Stat1, Irf9*, *Isg15* and *Atg5* thereby inhibiting cellular antiviral signaling and autophagy. Consistent with these observations, ATF3-depleted cells showed enhanced antiviral responses and induction of robust autophagy. Furthermore, we show that JEV replication was significantly reduced in ATF3-depleted cells. Our findings identify ATF3 as a negative regulator of antiviral signaling and cellular autophagy in mammalian cells, and demonstrate its important role in JEV life cycle.

## Introduction

Viruses are arduous pathogens that pose a unique challenge to our immune system as they are composed of the host-derived molecules. However, viral nucleic acids possess unique features distinguishing them from the host which have possibly led to the evolution of Pattern Recognition Receptors (PRRs) for their detection. Among the PRRs, RIG-I-like receptors (RLRs) are ubiquitous cytosolic detectors which play an integral role in antiviral responses^1^. Following the detection of viral infections, the PRR-initiated antiviral signaling rapidly induces the production of type 1 interferons (IFNa and IFNb) and other pro-inflammatory cytokines. The IFNs once released into the extracellular milieu bind to their respective membrane-bound receptors and initiate downstream signaling leading to the modulation of expression of a cohort of antiviral genes termed as Interferon Sensitive Genes (ISGs)^2^. The IFNs can potentially act in an autocrine or paracrine manner to subvert an existing viral infection or induce a pre-emptive antiviral state, respectively. Both the primary response (PRR activation followed by IFN synthesis) and the secondary response (IFN- receptor interaction to modulate the ISG expression) are driven by a dedicated family of transcription factors (TFs). The primary response is mainly driven by Interferon Regulatory Factor (IRF) family of TFs while the secondary response depends on the activity of the STAT proteins as part of the JAK-STAT pathway^3, 4^. Binding of IFNs to their receptors leads to receptor dimerization followed by the activation of IRF and STAT family of transcription factors. STAT1 and STAT2 dimerize and interact with IRF9 to form the Interferon-Stimulated Gene Factor 3 (ISGF3) complex^5^. This complex then translocates to the nucleus and binds to the conserved Interferon-Stimulated Response Elements (ISREs) resulting in the induction of various ISGs. Apart from the induction of ISGs, type 1 IFN signaling plays a pivotal role in regulating other cellular processes like autoimmunity^6^, cancer^7^ and autophagy^8, 9^.

Autophagy is a highly conserved phenomenon in which cells digest their own cytoplasmic content in the lysosomes. The term autophagy refers to the collection of various cellular processes including macroautophagy, microautophagy, chaperone-mediated autophagy and non-canonical autophagy. Macroautophagy is the major route for degradation of cytoplasmic constituents where cellular components are sequestered within a double-membrane structure called autophagosome, followed by its fusion with lysosomes. Autophagy is a tightly regulated phenomenon and its dysregulation results in various diseases. It has been reported that autophagy can be regulated at both transcriptional and translational levels. Initially, Tor was shown to regulate autophagy in yeast^10^. It was reported that nutrient deprivation leads to phosphorylation of TORC1 resulting in the inhibition of autophagy^11^. Apart from TORC1, several transcription factors have also been shown to regulate autophagy. It was observed that starvation leads to the phosphorylation and activation of FOXO3 which then promotes autophagy via regulating family of ATG genes^12, 13^. Apart from FOXO family of transcription factors, autophagy has been shown to be regulated by other transcription factors including E2F, NFKB and TP53. E2F family of transcription factors were shown to regulate autophagy directly via binding to some of the key autophagy genes like *Lc3*, *Atg1* and *Dram* ^14, 15^. NFKB family of the transcription factor are well characterized for their role in inflammation. However, it was shown that there was an inverse relation between autophagy and NFKB and both regulated each other positively. It was shown that while IKK complex leads to the induction of autophagy, functional autophagy was required for the activation of NFKB^16, 17^. TP53, a well-known tumor suppressor, was shown to regulate autophagy in a dual manner depending on its location inside the cells. Its nuclear location leads to the activation of autophagy whereas its cytoplasmic location leads to suppression of autophagy^18, 19^. Apart from regulation of autophagy by above-mentioned transcription factors, a strong interplay between ATF3 and autophagy was recently reported where ATF3 was shown to regulate autophagy via beclin1 on the one hand, whereas on the other hand, autophagy was shown to influence nuclear translocation of ATF3^20, 21^.

Activating Transcription Factor 3 (ATF3) belongs to the ATF/**c**AMP Responsive Element-Binding (CREB) family of TFs and is known to be induced during inflammation and genotoxic stress^22–24^. ATF3 was shown to be induced by lipopolysaccharides (LPS) and regulate TLR4 signaling via epigenetic regulation. Furthermore, it was shown that ATF3 interacts with HDAC1 thereby causing histone deacetylation and repression of the *Il6* and *Il12b* promoter^22^. Besides being a negative regulator of inflammatory responses, ATF3 has been shown to positively regulate various cellular pathways suggesting that it can act either as an activator or repressor of transcription^25, 26^. Apart from the various TLR ligands, ATF3 has also been shown to be induced by High-Density Lipoprotein (HDL), thus providing mechanistic insights into anti-inflammatory nature of HDL^27^. ATF3 has also been shown to play an important role in inhibition of other cellular responses including the inhibition of allergen-induced airway inflammation in the mouse model of human asthma^28^. These studies thus suggest that ATF3 can be induced by diverse pathways and act as a negative regulator of inflammation.

Apart from regulating inflammatory responses, ATF3 has been recently shown to modulate cellular antiviral signaling and autophagy^20, 29^. It is well established that viral infections lead to induction of primary and secondary type I IFN signaling. Recently, a role of type I IFNs in the induction of autophagy was also reported^8, 9^. However, modulation of cellular antiviral signalling and autophagy in the absence of functional type I IFNs has not been studied. Here, we have characterized the role of ATF3 in the regulation of cellular antiviral and autophagy signaling in neuronal cells, which we show are devoid of type I IFNs. Furthermore, we have characterized the role of ATF3 in the life cycle of Japanese encephalitis virus (JEV), an RNA genome containing neurotropic flavivirus, that has been shown to signal via RIG-I^30^. We observed that JEV infection of mammalian cells leads to a robust induction of ATF3. We demonstrate that ATF3 acts as negative regulator of cellular antiviral and autophagy signalling pathways. We further show that ATF3 binds to the promoter region of *Stat1*, *Irf9, Isg15 and Atg5* thereby uncovering a novel mechanism showing a direct role of ATF3 in the regulation of the antiviral responses and autophagy in the absence of type I IFN signalling in JEV-infected neuronal cells.

## Materials and Methods

### Cell lines, virus, and antibodies

Mouse neuroblastoma cells (Neuro2a), porcine kidney stable cells (PS), human embryonic kidney cells (HEK293), and human cervical epithelial cells (HeLa) were obtained from the National Centre for Cell Sciences, Pune and maintained in DMEM (Invitrogen) supplemented with 10% foetal bovine serum (FBS), penicillin/streptomycin and 2 mM glutamine. The P20778T strain of JEV grown in PS cells was used and titrated by plaque formation on PS cells^31^. The P20778 strain of JEV was used in our studies. Rabbit polyclonal anti-ATF3 antibody (Cat. No. sc-188X) and anti-STAT1 antibody (Cat. No. sc-346) were obtained from Santa Cruz, and rabbit polyclonal anti-JEV NS3 antibody was made in house. Rabbit monoclonal anti-GAPDH antibody (Cat. No. 2118), anti-LC3 antibody (Cat no 3868), anti-ATG5 antibody (Cat. No. 12994) and rabbit IgG (Cat. No. 2729) were procured from Cell Signalling Technology.

### Microarray and ChIP-Seq data analysis

To identify a putative genome-wide role of ATF3, we integrated previously published whole genome occupancy profiling (ChIP-Seq) and transcriptome profiling (microarray) data. ChIP-Seq (ATF3) and microarray data in WT and ATF3 KO was obtained from Gene Expression Omnibus (GEO) (IDs: ChIP-Seq: GSE36104; microarray: GSE32574). We used GEO2R utility to find out differentially expressed genes in KO condition over WT. Also, we obtained the ATF3 binding sites from ChIP-Seq data (as submitted post peak calling on GEO) and identified the genes with a binding signal in +/− 10 kb of gene promoters. Also, we used the sequence of binding sites to predict the binding motif of ATF3 using STEME^32^ and then scanned the obtained motif to identify other possible binding sites of ATF3. After that, we intersected the two gene lists to identify genes that could be potentially regulated by ATF3.

### siRNA transfection

ATF3 siRNA (Cat. No. L-058604) or non-targeting control siRNA (Cat. No. D-001810-10-20) obtained from Dharmacon (ON-TARGET plus SMART pool) were transfected at a final concentration of 20 nM using DharmaFECT 1 following the manufacturers’ protocol. Briefly, 20 nmol siRNA was complexed with 5 µl transfection reagent which was then added to the cell monolayer in a six-well plate in the presence of DMEM with 10% FBS.

### Quantitative Real Time PCR (qRT PCR)

Total RNA from cells was isolated using RNeasy kit (Qiagen) with in-column DNase digestion. Two hundred ng of total RNA was reverse-transcribed using random hexamers and ImProm-II reverse transcription system (Promega). All qPCR were performed using 2x Fast SYBR-Green mix (Invitrogen) in ABI 7500 Fast RT-PCR machine (Applied Biosystems). For all experiments, *Gapdh* levels were used for normalization. List of primers used for quantifying the various gene transcripts is provided (Table 1).

**Table 1:** Nucleotide sequence  of the primers used in the study.

Gene Name	Forward Primer	Reverse Primer
Atf3	GAGGATTTTGCTAACCTGACACC	TTGACGGTAACTGACTCCAGC
Isg15	GGTGTCCGTGACTAACTCCAT	TGGAAAGGGTAAGACCGTCCT
Isg20	TGGGCCTCAAAGGGTGAGT	CGGGTCGGATGTACTTGTCATA
Mx1	GACCATAGGGGTCTTGACCAA	AGACTTGCTCTTTCTGAAAAGCC
Mx2	GAGGCTCTTCAGAATGAGCAAA	CTCTGCGGTCAGTCTCTCT
Gbp1	ACAACTCAGCTAACTTTGTGGG	TGATACACAGGCGAGGCATATTA
Ifih1	AGATCAACACCTGTGGTAACACC	CTCTAGGGCCTCCACGAACA
Oas1a	GCCTGATCCCAGAATCTATGC	GAGCAACTCTAGGGCGTACTG
Stat1	TCACAGTGGTTCGAGCTTCAG	GCAAACGAGACATCATAGGCA
Stat2	TCCTGCCAATGGACGTTCG	GTCCCACTGGTTCAGTTGGT
Irf1	ATGCCAATCACTCGAATGCG	TTGTATCGGCCTGTGTGAATG
Irf2	AATTCCAATACGATACCAGGGCT	GAGCGGAGCATCCTTTTCCA
Irf3	GAGAGCCGAACGAGGTTCAG	CTTCCAGGTTGACACGTCCG
Irf9	GCCGAGTGGTGGGTAAGAC	GCAAAGGCGCTGAACAAAGAG
Tnfsf10	ATGGTGATTTGCATAGTGCTCC	GCAAGCAGGGTCTGTTCAAGA
Ddx60	TTCCACTGCCCAAAATAGGAAAA	GCCAGCAACATGAGTCTTAGGAT
Dhx58	GGAAGTGATCTTACCTGCTCTGG	TTGCCTCTGTCTACCGTCTCT
Aim2	GTCACCAGTTCCTCAGTTGTG	CACCTCCATTGTCCCTGTTTTAT
Nlrp3	ATTACCCGCCCGAGAAAGG	TCGCAGCAAAGATCCACACAG
Trim25	ATGGCTCAGGTAACAAGGGAG	GGGAGCAACAGGGGTTTTCTT
Trim66	CTTTGCCTTGTACTGCCCTCT	TGGATTTCTTATGTGCCACCTG
Jak2	TTGTGGTATTACGCCTGTGTATC	ATGCCTGGTTGACTCGTCTAT
Adar	TGAGCATAGCAAGTGGAGATACC	GCCGCCCTTTGAGAAACTCT
Bst2	TGTTCGGGGTTACCTTAGTCA	GCAGGAGTTTGCCTGTGTCT
Casp1	ACAAGGCACGGGACCTATG	TCCCAGTCAGTCCTGGAAATG
H2-M3	ACATTCACTGCGCTATTTCCA	CCTCGGAATTTCCTCAATGCT
Ifi30	CCTGGTCTCCGATCCTACCAT	TTGCAGGTGGTTGTGCCTT
Ifitm2	TGGGCTTCGTTGCCTATGC	AGAATGGGGTGTTCTTTGTGC
Ifitm3	CCCCCAAACTACGAAAGAATCA	ACCATCTTCCGATCCCTAGAC
Ifit1	CTGAGATGTCACTTCACATGGAA	GTGCATCCCCAATGGGTTCT
Ifit2	AGTACAACGAGTAAGGAGTCACT	AGGCCAGTATGTTGCACATGG
Pkr	ATGCACGGAGTAGCCATTACG	TGACAATCCACCTTGTTTTCGT
Ddx3x	CAGAGTGGAGGAAGTACAGCA	TCACCCCGTGATCCAAAACTG
Tbk1	ACTGGTGATCTCTATGCTGTCA	TTCTGGAAGTCCATACGCATTG
Ticam1	AACCTCCACATCCCCTGTTTT	GCCCTGGCATGGATAACCA
Traf3	CAGCCTAACCCACCCCTAAAG	TCTTCCACCGTCTTCACAAAC
Traf6	AAAGCGAGAGATTCTTTCCCTG	ACTGGGGACAATTCACTAGAGC
Ifna4	Sigma Kiqstart primer (M2_Ifna4)	
Ifnb1	Sigma Kiqstart primer (M1_Ifnb1)	
ATF3	CCTCTGCGCTGGAATCAGTC	TTCTTTCTCGTCGCCTCTTTTT
JEV	AGAGCACCAAGGGAATGAAATAGT	AATAAGTTGTAGTTGGGCACTCTG
Eif2ak3	AGTCCCTGCTCGAATCTTCCT	TCCCAAGGCAGAACAGATATACC
Ppp1r15a	GAGGGACGCCCACAACTTC	TTACCAGAGACAGGGGTAGGT
Slc7a5	ATATCACGCTGCTCAACGGTG	CTCCAGCATGTAGGCGTAGTC
Ero1l	TTCTGCCAGGTTAGTGGTTACC	GTTTGACGGCACAGTCTCTTC
Cebpg	TCGGATCACATTGCTCTGATTTC	TGTGCCTGAGTATGAATGACACT
Atf6	GACTCACCCATCCGAGTTGTG	CTCCCAGTCTTCATCTGGTCC
Canx	ATGGAAGGGAAGTGGTTACTGT	GCTTTGTAGGTGACCTTTGGAG
Dnajc3	GGCGCTGAGTGTGGAGTAAAT	GCGTGAAACTGTGATAAGGCG
Atg101	GGAGGTGTGGACTGTCAAGG	CCGTGTCAAACACGTTGTCC
Atg12	TAAACTGGTGGCCTCGGAAC	ATCCCCATGCCTGGGATTTG
Atg13	TTGTCCGAAAAGTGGGAGCA	CAGCATCCTCCAGCTCCAAA
Atg14	GTGGCGAAAACCTCAGCAAG	TTCAAAGGGTCCTGACCTGC
Atg16l2	ACAGGTGTTCAGGGCAGATG	CATTAACAGCAGTGCAGTGGG
Atg3	ACCACTGTCCAACATGGCAA	TACCCATCCCCCATCACCAT
Atg4b	TGGACGCAGCCACTTTGACAT	TAGGGCCAGTTCCCCCAATA
Atg4c	TTGGTTTGGAGATTCCCCCG	AAAATGTGAGCAACCACCGC
Atg5	ATCCAAGGATGCGGTTGAGG	ATCCAGAGCTGCTTGTGGTC
Atg9a	CCATCCTGGTCATTGCTGGT	ATGGAAGGGCAGACATTGGG
Atg9b	TGCGCTACACCAACTACCAG	GCGGAAGAGTGAGAAGGGAC
Eif4ebp1	GGGGACTACAGCACCACTC	GTTCCGACACTCCATCAGAAAT
Ppp4r1	GACGCGGACGGACTTGATG	CGTTCTCACTTGCAGCATACT
Ulk2	AGCTTCAGCATGAAAACATCGT	CGATTGGCATAAGACAACAGGA
Atg2b	GTCCCCTTGGACAAATGGTGT	GGACGGACAGGGAAATGGA

### Chromatin Immunoprecipitation (ChIP)

Mock-infected or JEV-infected Neuro2a cells (MOI 5) were trypsinized, washed and resuspended in 1 ml DMEM containing 10% FCS. Cells were fixed by adding 135 µl formaldehyde and incubation at 37 °C for 10 min followed by the addition of 500 µl glycine (1.25 M) for 5 min. Following the cross-linking, cells were lysed, and chromatin was sheared using bioruptor (Diagenode). The complexes were then incubated overnight with 5 µg ATF3 rabbit antibody and protein AG sepharose beads (20 µl). For the pull-down negative control, rabbit IgG was used. The beads were then washed extensively and incubated at 70 °C for 15 min for reversal of cross-linking. DNA was then purified manually using the chloroform-phenol based method. For the PCR amplification of the *Stat1* promoter sequence the primers used were CTTCTTGCAGGCTTGGTTGAC (forward primer, FP) and GCGGGATTCAGAATTGGGGA (reverse primer, RP); for the *Irf9* promoter PCR the primers were TTTCAGCGGCTCAGGTAAGA (FP) and GAGCTGAAGAAATGGGCAGG (RP); for the *Isg15* promoter PCR the primers were ACATCACTGGCACCATGACA (FP) and AGACAGCCACTTGTCTCCTC (RP); for the *Ifit1* promoter the PCR primers used were AGCCCCACTGTCTGTAGTTC (FP) and TGGGTCAGTGGTTAAGAGCA (RP); for the *Atg5* promoter the PCR primers used were GAGCAACTCAGGTCTTGCCA (FP) and CTCGGAACCAGAGTGAACCG (RP); for the *Atg101* promoter the PCR primers used were GACGCACACATGGGATGACA (FP) and GGCTCTGGACTGAAGCACTC (RP); and for *Atg14* promoter PCR primers used were AGTGCTGGCAGTGTGACTTG (FP) and GGGAACAGAAGTAAAGCCGGA (RP). A negative control was employed to rule out the enrichment of DNA due to the non-specific binding to beads. For this, the following primers were designed on mouse gene desert on chromosome 5: GATTGCAGAGTAAGATCCCTTGAT (FP) and GCGTAAGTTCTACATGCTGCTTTA (RP). One tenth of the input lysate employed for the pull-down was used as a positive control. The expected PCR product from *Stat1, Irf9, Isg15, Ifit1, Atg5, Atg101, Atg14* and the gene desert control was 236-, 260-, 283-, 206-, 142-, 150-, 115- and 124-bp, respectively.

### Statistical analysis

Statistical analysis was performed using the Student’s t-test. The difference was considered significant at p < 0.05 and is indicated in the figures as *p < 0.05; **p < 0.01; ***p < 0.001.

## Results

### ATF3 is induced in mammalian cells following JEV infection

**Figure 1:**
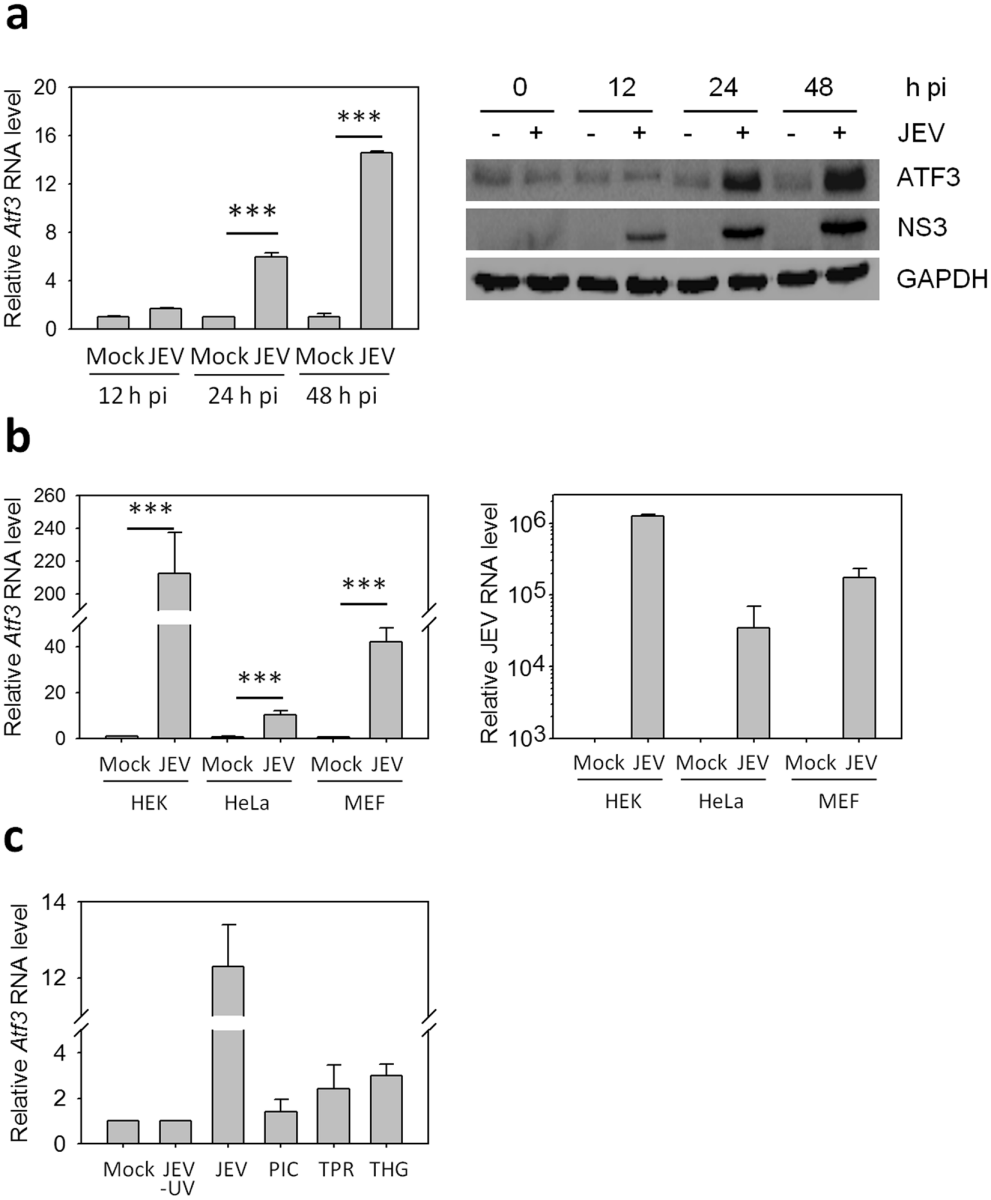
ATF3 induction in mammalian cells during JEV infection. (**a**) Neuro2a cells were mock-infected or infected with JEV (MOI 5) and at different times post-infection (pi) cells were harvested to isolate total RNA and proteins. qRT PCR was performed to quantitate the *Atf3* expression. Relative abundance of *Atf3* transcript at various time points is shown (left panel). The cell lysates were Western blotted to study the relative amounts of ATF3 following the virus infection that was monitored by the presence of the JEV NS3 protein (right panel). (**b**) Mammalian cells were mock-infected or infected with JEV (MOI 5). Relative levels of *Atf3* transcripts were determined by qRT PCR in various cell lines 24 h pi (left panel). The infection of cells with JEV was established by qRT PCR for JEV genomic RNA (right panel). (**c**) Neuro2a cells were incubated with UV-inactivated JEV (JEV-UV) or infected with JEV (MOI 5), or transfected with 1 µg poly(IC) (PIC) or triphosphate RNA (TPR), or treated with 1 µM thapsigargin (THG). Total RNA was isolated from these cells 24 h later, and qRT PCR was performed to quantitate the *Atf3* expression. The relative abundance of *Atf3* transcript is shown after various treatments. The *Gapdh* levels were used for normalization. All the experiments were performed in triplicates on three different occasions. The statistical significance of the difference between the data was established by Student’s t-test; *p < 0.05; **p < 0.01; ***p < 0.001.

ATF3 is known to be induced under the conditions of stress. Accordingly, we sought to study the transcriptional status of *Atf3* during JEV infections. We found that *Atf3* transcript was highly induced (>10-folds) following JEV infection of mouse neuronal cells (Neuro2a) and the levels of ATF3, as validated by western blotting, increased as the virus replication progressed (Fig. 1a). The JEV-mediated induction of *Atf3* was not cell-specific as similar observations were made in diverse cells like human embryonic kidney (HEK) cells, human cervical epithelial (HeLa) cells, and mouse embryo fibroblast (MEF) cells (Fig. 1b). It may, however, be noted that the extent of ATF3 induction following the virus infection varied greatly among the different cell lines perhaps due to the susceptibility of different cells to JEV infection.

ATF3 induction following JEV infection could be through multiple pathways including the TLR, RLR and UPR pathways^24^. We observed ~2-fold induction of ATF3 (Fig. 1c) when Neuro2a cells were treated with poly(IC) (induces TLR pathway), triphosphate RNA (induces RLR pathway) as well as Thapsigargin (induces UPR pathway). While the levels of ATF3 were elevated >10-fold in JEV-infected Neuro2a cells, UV-inactivated JEV failed to induce ATF3 synthesis. This suggested that JEV replication was necessary and various signalling pathways might have an additive effect towards the induction of ATF3 during the viral infection.

### ChIP-Seq and microarray data identifies antiviral genes potentially regulated by ATF3

We utilized computational biology approach to gain an insight into the novel pathways that might be regulated by ATF3. Analysis of the ChIP-Seq data from mouse dendritic cells^33^ identified 12154 unique DNA sequences that could potentially bind ATF3. A microarray-based study had identified a set of gene transcripts that were up-regulated in *Atf3* knock-out (KO) mouse BMDM cells when compared with wild-type (WT) cells^34^. To obtain the targets of ATF3, we overlaid the ATF3 ChIP-Seq data with the microarray data on the premise that this analysis may lead us to genes regulated by ATF3. We found 17 genes that showed ATF3 binding in ChIP-Seq study and were up-regulated in *Atf3* KO cells (Table 2). A review of the known functions of these genes revealed a role in inflammation for many of them, with some of them known to be ISGs (for example, *Ch25h, Rsg2*, and *Gbp8*) having a demonstrated antiviral role^35, 36^. Hypothesizing that ATF3 might be a regulator of the antiviral function; we scanned the promoter region of various ISGs *in-silico* which revealed that many ISGs had putative ATF3 binding sites in their promoter region (Table 3) suggesting that ATF3 might regulate the antiviral function through a direct regulation of the various ISGs.

**Table 2:** Genes predicted to be regulated by ATF3.

Gene	Function	Reference
*Slc7a11	amino acid transport, role in inflammation	51
*Mgl2*	possible role in antigen uptake	52
*Asns*	synthesis of asparagine	53
*Ttc39c*	function not known	
*Sgk1	protein kinase, role in cellular stress response and differentiation of T cells	54
*Sars*	a class II amino-acyl tRNA ligase family member	55
*Gdf15	a TGF-beta family member	56
*Rgs1	a member of the regulator of G-protein signalling family and regulates T cells trafficking	57
*Psph*	a member of the family of phosphotransferases	58
*Gbp8	regulates type I IFN responses	59
*Tmem171*	function not known	
*Pla1a	a phospholipase, involved in Hepatitis virus assembly	60
*Ch25h	involved in cholesterol and lipid metabolism, has an antiviral function	49
*Aqp9	a member of the aqaporin family of proteins, induced under inflammatory conditions	61
*Rgs2	a member of the regulator of G-protein signalling family, regulates antiviral immunity	62
*Ncapg2*	a member of the Condensin2nSMC family of proteins	
*Rgs18	a member of the regulator of G-protein signalling family, role in TLR signaling	63

An in-house python program was written to analyze the published microarray and ChIP data^33, 34^ to predict genes that may be regulated by ATF3. Known or predicted functions of the gene have been listed, and genes with a known role in either inflammation or antiviral immunity are marked with an asterisk.

**Table 3:** Location of the putative ATF3 binding sequence in the promoter region of the various ISGs.

Gene Name	Putative ATF3 binding site/s
*Adar*	−166
*Aim2*	−1628
*Ddx58*	−2306
*Ddx60*	−1844
*H2-M3*	−4153
*Ifit1*	−937
*Irf9*	+396
*Isg15*	−128, −1791, −1870, −1880
*Stat1*	−450
*Stat2*	−1451
*Ticam1*	−1166
*Tnfsf10*	−2523

The −5 kb to +1 kb region of the gene containing the putative promoter elements was scanned for the ATF3 binding motif (TGACGTCA) using an in-house written python script. The position of putative ATF3 binding sites relative to the transcription start site in the various ISGs is listed.

### ATF3 negatively regulates various ISGs

**Figure 2:**
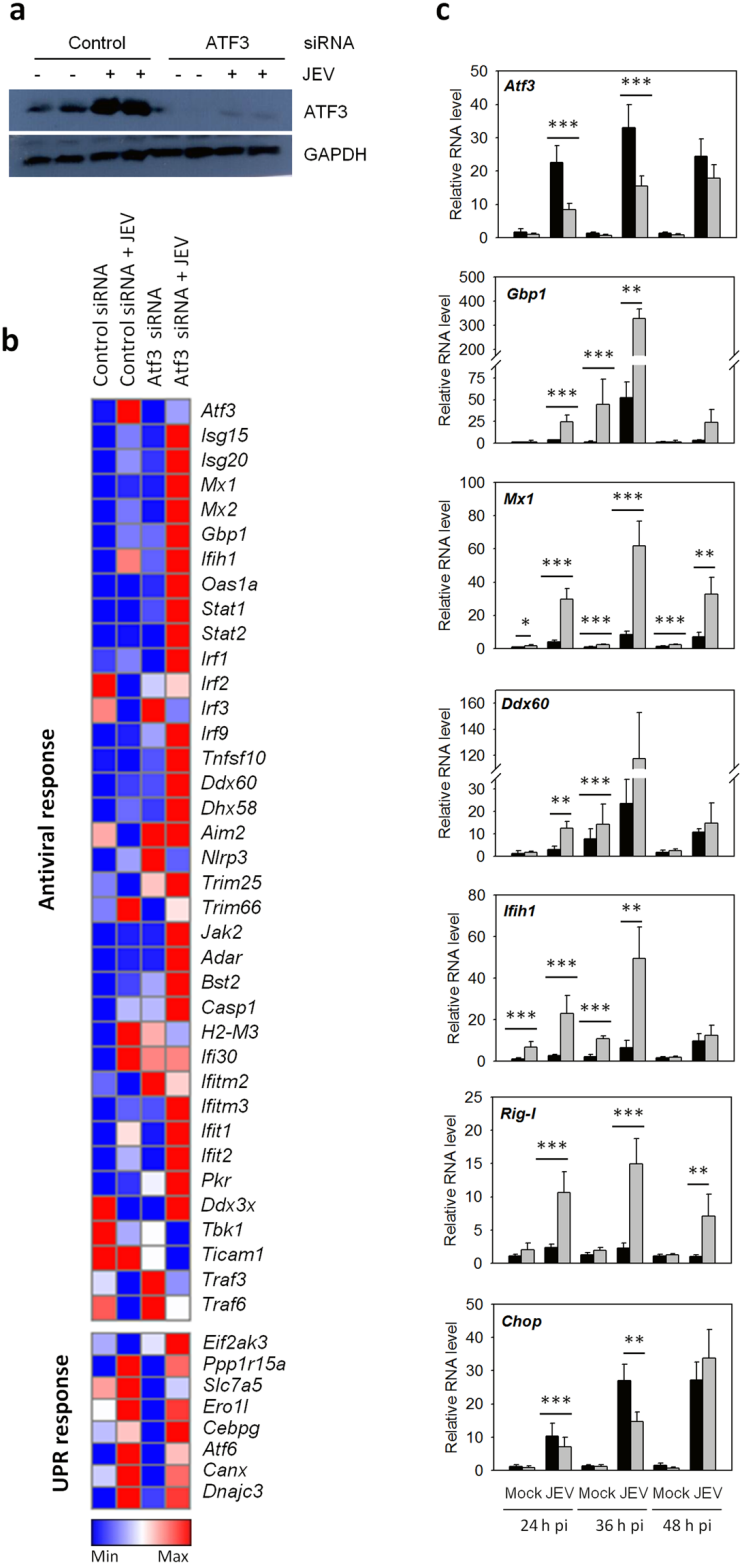
ATF3 acts as a negative regulator of the antiviral response. (**a**) Neuro2a cells were treated with either a control non-targeting siRNA or the *Atf3*-specific siRNA for 48 h and then infected with JEV (MOI 5). Total proteins from the cell lysates harvested at 24 h pi were Western blotted to establish the ATF3 depletion. (**b**) Total RNA was isolated from the cells at 24 h pi and qRT PCR performed in triplicates to determine the relative transcript levels for various ISGs or members of UPR pathway. Mean values were used to create the heat map demonstrating the gene-expression profiles using the Gene-E software. (**c**) Neuro2a cells were treated with either a control non-targeting siRNA or the *Atf3*-specific siRNA for 48 h and then infected with JEV (MOI 5) for different time points. The experiment was done in triplicates on three different occasions. Relative expression of various genes at different times pi was studied by qRT PCR performed in triplicates for each sample, and the mean is plotted. Transcript level in control siRNA-treated cells is shown by the black bar while that in the *Atf3* siRNA-treated cells is shown by the gray bar. The *Gapdh* levels were used for normalization. The statistical significance of the difference between the data was established by Student’s t-test; *p < 0.05; **p < 0.01; ***p < 0.001.

Since robust ATF3 induction was observed during JEV infection, we sought to study its role in cellular antiviral responses. The levels of various ISGs were studied post-JEV infection in Neuro2a cells where ATF3 levels had been knocked-down by siRNA (Fig. 2a). The siRNA transfections were carried out in the presence of FBS, and cell viability was >95% as seen by Trypan blue staining, suggesting that siRNA transfection had no toxic effect. Out of the 36 antiviral genes studied, 25 showed a significantly increased (≥2.5-fold) transcript level in *Atf3* siRNA-treated JEV-infected cells compared to control siRNA-treated cells. However, none of the genes involved in the Unfolded Protein Response (UPR) pathway were affected suggesting that ATF3 specifically regulated the cellular antiviral pathway. Interestingly, levels of some of the antiviral genes such as *Isg15, Mx1, Gbp1, Ifih1, Irf9, DDx60, Nlrp3, Bst2, Ifit1, Rig-I* and *Casp1* were significantly up-regulated (≥2.5-fold) in ATF3-depleted uninfected cells, suggesting that ATF3 might be regulating the basal levels of these antiviral genes (Fig. 2b). We then studied the expression of some of the ISGs in Neuro2a cells following JEV infection at different time points. In agreement with our previous findings, these data suggested that ATF3 negatively regulated the ISGs (Fig. 2c). ATF3 is known to positively regulate the *Chop/Ddit3* transcript^37^. In agreement with the published data, our study showed a reduced level of this transcript in ATF3-depleted cells (Fig. 2c), thus validating our experimental setup and findings.

### ATF3 depletion induces *Stat1, Stat2* and *Irf9* transcripts

ATF3 has been shown to directly repress the *Ifnb1* promoter^38^. It can negatively regulate the expression of IFNs^29^, thereby controlling the expression of various ISGs involved in antiviral function. Since neuronal cells are known to be deficient for IFN signalling^39^, an alternate mechanism must operate in these cells for ATF3-mediated suppression of antiviral genes.

**Figure 3:**
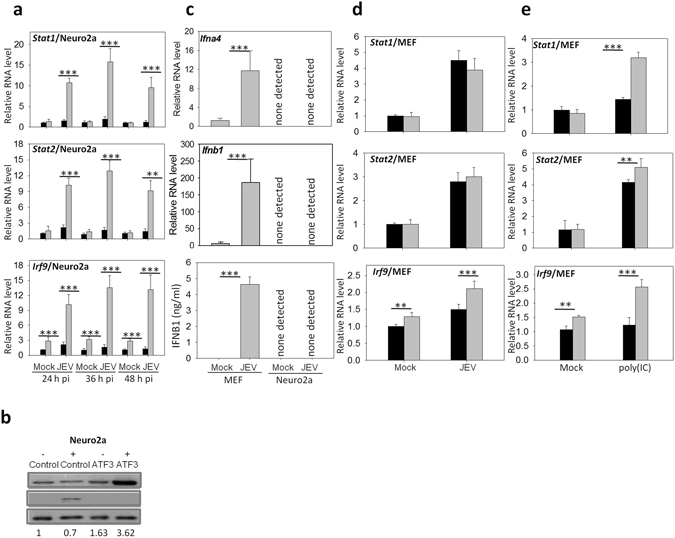
ATF3 negatively regulates tyvpe I interferon system. (**a**) Neuro2a cells were treated with either a control non-targeting or the *Atf3*-specific siRNA for 48 h and then infected with JEV (MOI 5) for different time points. qRT PCR was done to quantify the relative levels of STAT and IRF family gene transcripts in control siRNA-treated cells (black bars) or the *Atf3* siRNA-treated cells (grey bars). (**b**) Lysates from siRNA-treated JEV-infected (+) and mock-infected (−) cells were western blotted. Cells treated with control siRNA when infected with JEV showed a high level of ATF3 expression seen as a prominent band. Shown below is the ratio of STAT1/GAPDH. On a longer exposure of the blot the two isoforms of STAT1 could be seen (not shown here). (**c**) Neuro2a or MEF cells were mock-infected or infected with JEV (MOI 5). At 24 h pi qRT PCR was done to quantify the relative levels of *Ifna4* and *Ifnb1* transcripts in the cells. These transcripts were not detected in Neuro2a cells whereas they were upregulated in MEF cells following the infection. The lowest panel shows the levels of IFNb1 in the culture soup. IFNb1 could not be detected in Neuro2a cells whereas its levels were upregulated in MEF cells following JEV infection. (**d**) MEF cells were treated with control siRNA (black bars) or *Atf3-*specific siRNA (grey bars) and then infected with JEV. At 24 h pi qRT PCR was done to quantify the relative levels of *Stat1, Stat2* and *Irf9* transcripts. (**e**) MEF cells were treated with control siRNA (black bars) or *Atf3-*specific siRNA (grey bars) and then transfected with 1 μg/ml poly(IC). At 24 h post-transfection, qRT PCR was done to quantify the relative levels of *Stat1, Stat2* and *Irf9* transcripts. All the experiments were done in triplicates on three different occasions. The *Gapdh* levels were used for normalization. The statistical significance of the difference between the data was established by Student’s t-test; *p < 0.05; **p < 0.01; ***p < 0.001.

STAT and IRF represent the family of TFs that regulates the expression of genes involved in primary and secondary immune responses, respectively. Many of the ATF3-repressed genes described above are known to be under the regulation of TFs from these families. Therefore, it is possible that the enhancement of gene expression observed upon *Atf3* silencing is an indirect effect of up-regulation of genes from these TF families. We tested the transcript levels of multiple genes from STAT (*Stat1, Stat2, Stat3, Stat4, Stat5a, Stat5b, Stat6*) and IRF family (*Irf1, Irf3, Irf9*) and found that only *Stat1, Stat2 and Irf9* showed up-regulation in ATF3-depleted Neuro2a cells infected with JEV (Fig. 3a). Interestingly, ATF3 was also found to regulate the basal levels of *Stat2* and *Irf9* in uninfected cells (Fig. 3a). STAT1 is a transcription factor which is a major regulator of cellular antiviral response. We, therefore, performed western blotting of STAT1 to corroborate the above finding on its transcript. Indeed, we found a 3.8-fold enhanced expression of STAT1 in ATF3-depleted Neuro2a cells infected with JEV (Fig. 3b).

It is well established that STAT1, STAT2 and IRF9 form the secondary response of type I IFN signalling which is initiated once extracellular IFNs bind to their cognate receptors. Following the induction, STAT1, STAT2 and IRF9 assemble to form the ISGF3 complex that binds to the ISG elements in the promoter region to regulate the expression of various antiviral genes. However, how the antiviral responses are regulated in cells devoid of type I IFN signalling needs further studies. There are conflicting reports about the ability of neurons to produce IFN. For example, 2.5–3% of the hippocampal neurons in mice infected with Theiler’s virus and La Crosse virus produced IFN^40^, whereas dorsal root ganglion neurons infected with herpes simples virus-1 failed to induce IFN mRNA^39^. We, therefore, sought to examine if Neuro2a cells produced IFN following the JEV infection. We found no *Ifna4* and *Ifnb1* transcripts or secreted IFNb1 in Neuro2a cells, and these were still not detected at 24 h after JEV infection. However, *Ifna4* and *Ifnb1* transcripts and secreted IFNb1 were found to be upregulated in MEF cells infected with JEV further confirming that neuronal cells are restricted in the production of type I IFNs (Fig. 3c). It can, thus, be speculated that in neuronal cells, which are deficient in induction of type 1 IFNs, ATF3 may directly modulate the antiviral gene expression through ISGF3.

We then checked the levels of *Stat1, Stat2 and Irf9* in IFN-sufficient, ATF3-depeleted MEF cells following JEV infection. We found that *Irf9* transcript was significantly induced following JEV infection; however, the transcript levels of *Stat1*and *Stat2* were not affected (Fig. 3d). This may be related to the inhibition of STAT1 activation by JEV^41^, and therefore we studied the transcript levels of the ISGF3 complex in the absence of ATF3 following the poly(IC) treatment. Here, we found that *Stat1, Stat2* and *Irf9* transcripts were significantly induced thereby supporting our hypothesis (Fig. 3e). The data presented here thus suggest that the negative regulation of the type 1 IFN response via the regulation of the ISGF3 complex may be mediated through ATF3.

### ATF3 binds to *Stat1* and *Irf9* promoter

**Figure 4:**
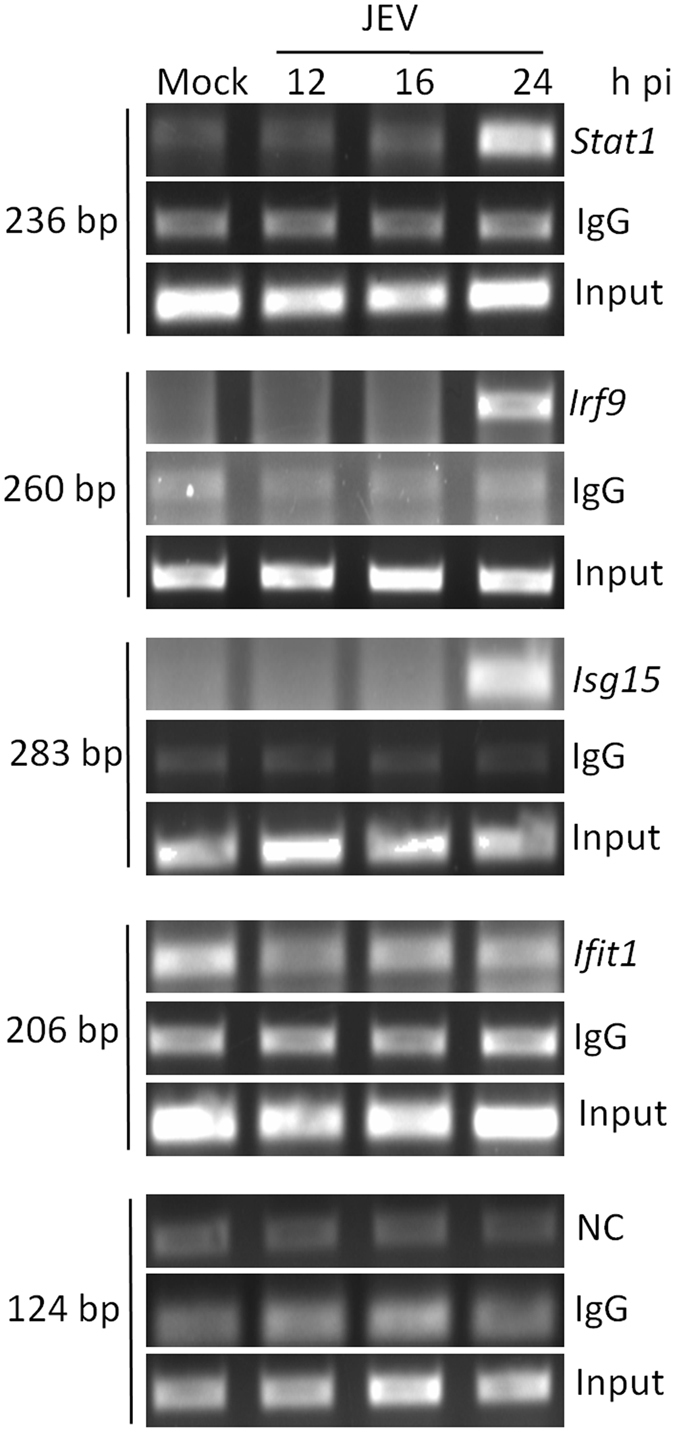
ATF3 binding to the promoter region of different antiviral genes. Neuro2a cells were mock-infected or infected with JEV (MOI 5) and at different time points chromatin immunoprecipitation was performed using ATF3-specific rabbit antibody or rabbit IgG (negative control for pull-down). Gene promoter-specific DNA was PCR amplified and separated on a 2% agarose gel. The relative abundance of pulled-down DNA during JEV infection as indicated by the PCR product is shown. The experiment was performed twice, and a representative figure is shown. NC indicates the negative control to rule out the enrichment of DNA due to the non-specific binding to beads. Here, oligonucleotide primers derived from the mouse gene desert were used. Ten percent of total lysate was used as input.

Since the loss of ATF3 led to the induction of transcripts of ISGF3 complex, we sought to investigate the molecular mechanism for the observed phenomenon. ATF3 has been shown to bind the promoter region of its target gene thereby repressing it^22^. Analysis of promoter region had also revealed potential ATF3 binding sites in numerous ISGs suggesting a direct regulation of ISGs by ATF3 (Table 3). We, therefore, performed chromatin immunoprecipitation (ChIP) to investigate if ATF3 could bind to the promoter region of various ISGs. Compared to mock-infected cells at 24 h pi, *Stat1* and *Irf9* amplification product of 236- and 260-bp was clearly visible and enriched in JEV-infected cells when ATF3 antibody was used for the pull-down (Fig. 4). However, an irrelevant IgG (negative control) failed to pull-down the desired product. Furthermore, under the same conditions, *Ifit1* promoter having a putative ATF3 binding site (Table 3) showed no enhanced binding to ATF3, suggesting that ATF3 specifically occupied the promoter regions of *Stat1* and *Irf9*. These data clearly demonstrate that ATF3 binds the promoter sequences of *Stat1* and *Irf9;* thereby suggesting that it could regulate the type 1IFN responses by regulating the ISGF3 complex.

### ATF3 binds to *the Isg15* promoter

ATF3 could also modulate the antiviral effect by directly controlling the expression of some of the classical ISGs. We had predicted the putative ATF3 binding site/s in *Isg15* and a few other classical ISG promoters (Table 3). Compared to mock-infected cells at 24 h pi, the *Isg15* amplification product of 283-bp was clearly detected in JEV-infected cells when ATF3 rabbit antibody was used for the pull-down in the ChIP assay (Fig. 4). These data show that ATF3 indeed binds to *Isg15* promoter during the JEV infection of Neuro2a cells.

### ATF3 negatively regulates autophagy in the absence of type I IFNs

**Figure 5:**
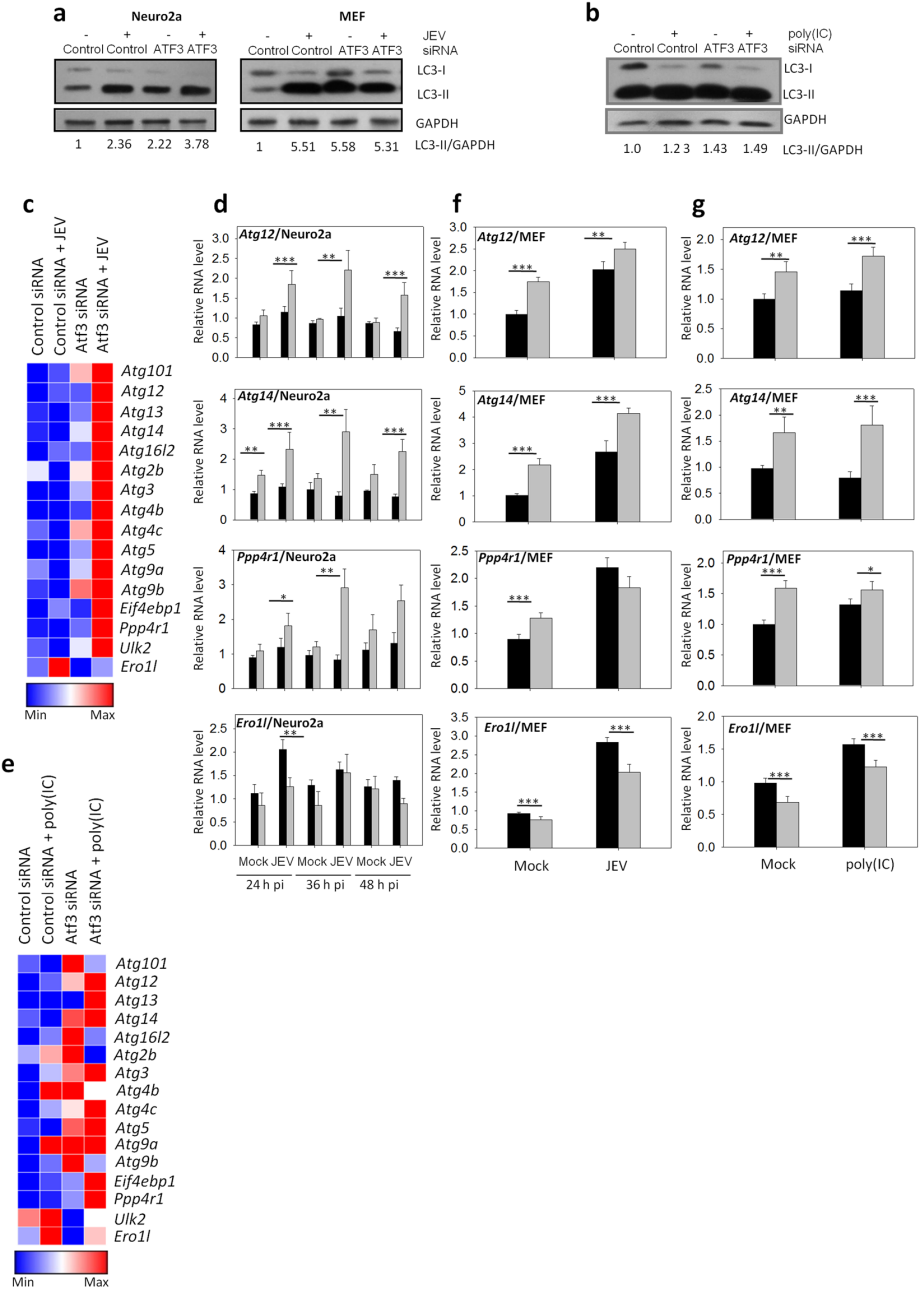
ATF3 negatively regulates cellular autophagy. (**a**) Neuro2a or MEF cells were treated with either a control or *Atf3*-specific siRNA for 48 h and then infected with JEV (MOI 5). Total proteins were harvested at 24 h pi as cell lysates. Western blotting of LC3 was performed to study the induction of cellular autophagy. Shown below is the ratio of LC3-II/GAPDH. (**b**) Neuro2a cells were treated with either a control or *Atf3*-specific siRNA for 48 h and then infected with JEV (MOI 5). Total RNA was isolated from the cells at 24 h pi and qRT PCR performed to determine the relative transcript levels for various autophagy-regulated genes. Mean values were used to create the heat map demonstrating the gene-expression profiles using the Gene-E software. (**d**) Neuro2a cells were treated with either a control or *Atf3*-specific siRNA for 48 h and then infected with JEV (MOI 5). Relative expression of various genes at different times pi was studied by qRT PCR, and the mean value is plotted. (**e**) MEF cells were treated with either a control or *Atf3*-specific siRNA for 48 h and then transfected with 1 μg/ml poly(IC). Total RNA was isolated from the cells at 24 h post-transfection, and qRT PCR performed to determine the relative transcript levels of various autophagy-regulated genes, and the mean values were used to create the heat map demonstrating the gene-expression profiles using the Gene-E software. (**f**) MEF cells were treated with either a control or *Atf3*-specific siRNA for 48 h and then infected with JEV (MOI 5) for 24 h. Relative expression of various autophagy-related genes was studied by qRT PCR, and the mean value is plotted. (**g**) MEF cells were treated with either a control or *Atf3*-specific siRNA for 48 h and transfected with 1 μg/ml poly(IC). Relative expression of various autophagy-related genes was studied 24 h later by qRT PCR and the mean value is plotted. Transcript level in control siRNA-treated cells is shown by the black bar while that in the *Atf3* siRNA-treated cells is shown by the gray bar. The *Gapdh* levels were used for normalization. All the experiments were done in triplicates on three different occasions.The statistical significance of the difference between the data was established by Student’s t-test; *p < 0.05; **p < 0.01; ***p < 0.001.

ATF3 induced in mice during cardiovascular stress was shown to regulate the autophagy^20^. We have recently shown that JEV infection induces autophagy in Neuro2a^42^. Importantly, a role for IFN I in the induction of autophagy in various mammalian cells has been reported^8^. It was, therefore of interest to study what role ATF3 might play in the induction of autophagy in Neuro2a cell shown to be deficient in IFN I synthesis. The effect of ATF3 depletion on JEV-induced autophagy was studied in Neuro2a by western blotting the LC3-II protein, a marker for autophagy. We observed that the loss of ATF3 led to an enhanced autophagy during JEV infection of Neuro2a as well as MEFs, thus negating an essential role of IFN I in ATF3-mediated autophagy in mammalian cells (Fig. 5a). This effect of ATF3 on autophagy was not specific to JEV infection since poly(IC)-induced autophagy in MEF cells was also affected in a similar manner by ATF3 (Fig. 5b). Interestingly, we observed a spontaneous induction of autophagy in ATF3-depleted cells pointing towards a robust regulation of autophagy via ATF3. These data show that ATF3 is a negative regulator of autophagy in mammalian cells.

To further understand the ATF3-mediated regulation of autophagy, expression of several of the autophagy-related genes was studied by the quantitative PCR of the RNA transcripts. ATF3 was found to regulate a battery of autophagy-related genes in Neuro2a cells in a negative manner (Fig. 5c). Expression of some of these autophagy-related genes was consistently enhanced in JEV-infected Neuro2a cells at different time points in ATF3-depleted cells (Fig. 5d). In IFN I sufficient MEF cells also ATF3 was found to downregulate the expression of autophagy-related genes in mock-treated, JEV-infected, or poly(IC)-treated cells (Fig. 5e–g). The repression of autophagy-related genes by ATF3 was a specific action as ATF3 depletion resulted in suppression of *Ero1l* gene that is involved in cellular UPR pathway but not known to have a role in autophagy. These data clearly established that ATF3 negatively regulated autophagy in cells by inhibiting the expression of autophagy-related genes.

### ATF3 binds to the Atg5 promoter

**Figure 6:**
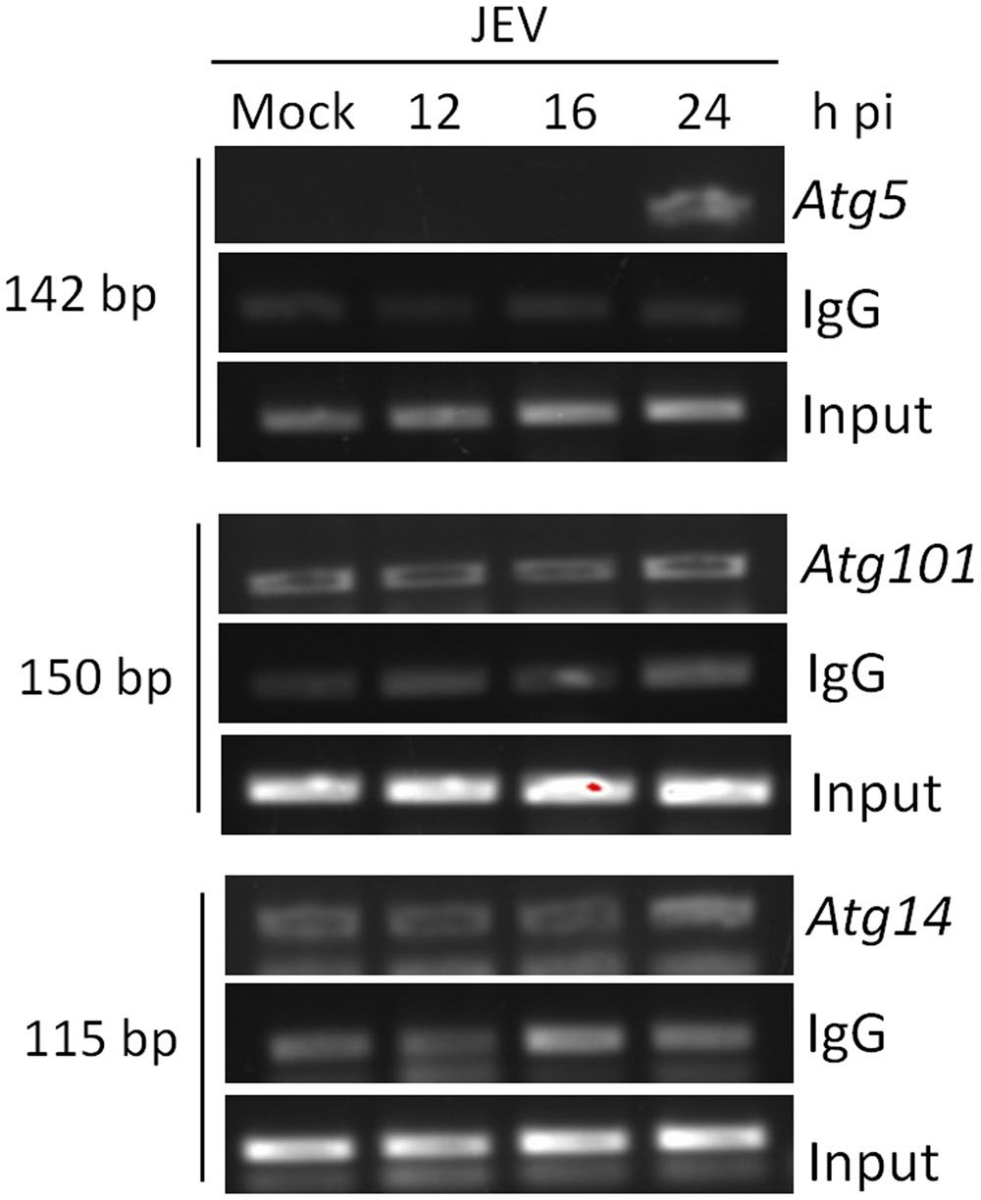
ATF3 binds to the promoter region of *Atg5*. Neuro2a cells were mock-infected or infected with JEV (MOI 5) and at different time points chromatin immunoprecipitation was performed using ATF3-specific rabbit antibody or rabbit IgG (negative control for pull-down). Gene promoter-specific DNA was PCR amplified and separated on a 2% agarose gel. The relative abundance of pulled-down DNA during JEV infection as indicated by the PCR product is shown. The experiment was performed twice, and a representative figure is shown. Ten percent of total lysate was used as input.

**Figure 7:**
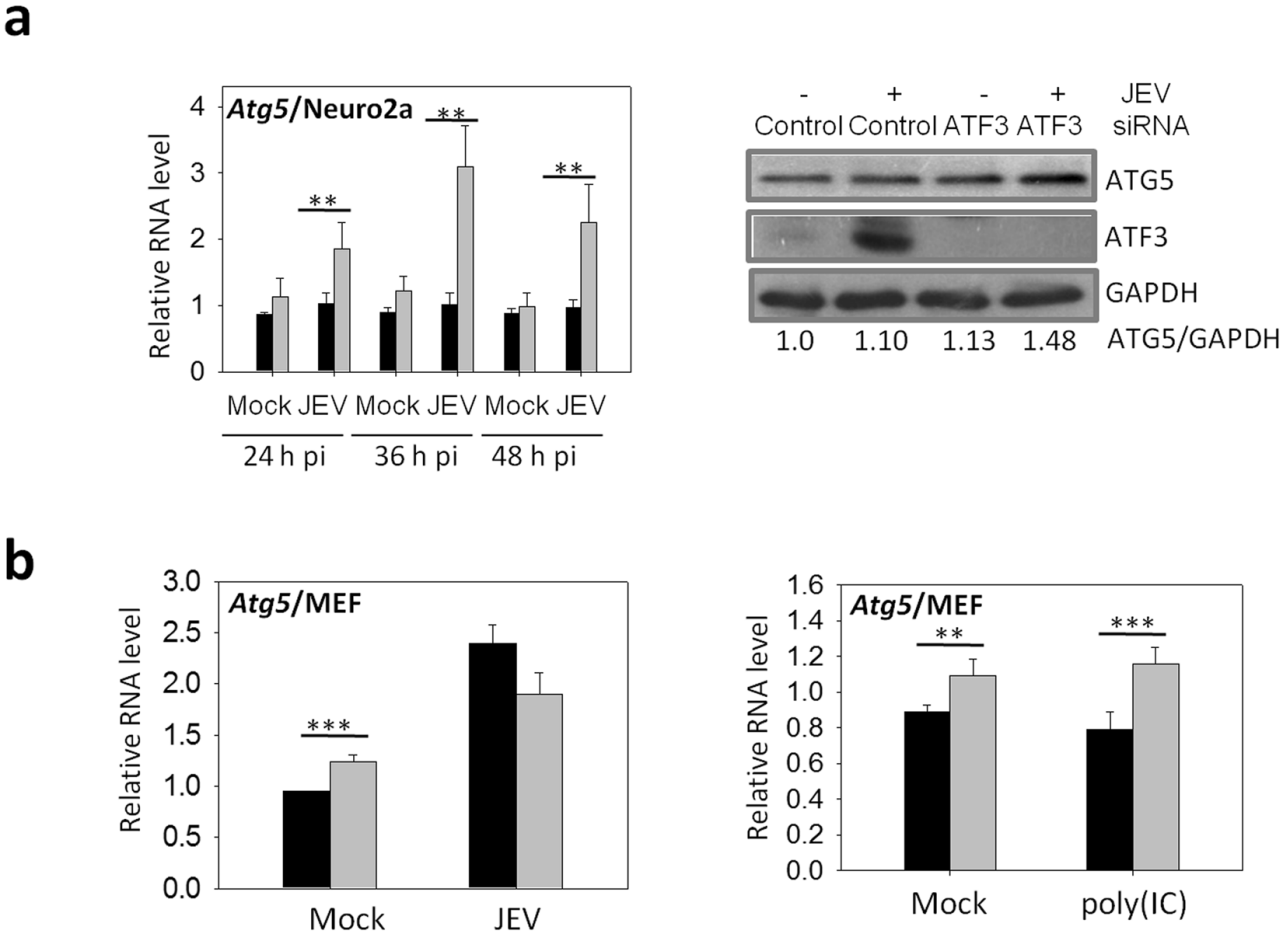
ATF3 negatively regulates *Atg5* gene. (**a**) Neuro2a cells were treated with either a control non-targeting siRNA or the *Atf3*-specific siRNA for 48 h and then infected with JEV (MOI 5). Relative expression of *Atg5* at different times pi was studied by qRT PCR, and the mean is plotted (left panel). Cell lysates were prepared 24 h pi, and Western blotted for ATG5 and ATF3. Shown below is the ratio of ATG5/GAPDH. (**b**) MEF cells were treated with either a control non-targeting siRNA or the *Atf3*-specific siRNA for 48 h and then infected with JEV (MOI 5) (left panel) or transfected with 1 μg/ml poly(IC) (right panel). Relative expression of *Atg5*, 24 h post-treatment, was studied by qRT PCR, and the mean is plotted. Transcript level in control siRNA-treated cells is shown by the black bar while that in the *Atf3* siRNA-treated cells is shown by the gray bar. The *Gapdh* levels were used for normalization. The experiments were done in triplicates on three different occasions. The statistical significance of the difference between the data was established by Student’s t-test; *p < 0.05; **p < 0.01; ***p < 0.001.

The data above showed a spontaneous induction of autophagy-related genes in the absence of ATF3, suggesting the possibility of direct control by the transcription factor ATF3. Scanning of the promoter region of several autophagy-related genes revealed putative ATF3 binding site(s) (Table 4). Indeed, ATF3 was found to bind specifically to the promoter region of *Atg5* in the ChIP assay. Thus, compared to mock-infected cells at 24 h pi, *the Atg5* amplification product of 142-bp was clearly visible and enriched in JEV-infected cells when ATF3 antibody was used for the pull-down (Fig. 6). However, an irrelevant IgG (negative control) failed to pull-down the desired product. Importantly, despite having predicted ATF3 binding site, we did not see an enrichment of *Atg101-* or *Atg14-*specific PCR product, suggesting that ATF3 specifically occupied the promoter regions of *Atg5*. These data show that ATF3 binds the *Atg5* promoter and could control its expression. Indeed, ATF3 was shown to significantly suppress the expression of *Atf5* transcripts in Neuro2a and MEF cells (Fig. 7). Accordingly, the level of ATG5 protein was enhanced in ATF3-depleted Neuro2a cells (Fig. 7a). These data clearly show that the autophagy-related gene *Atg5* is a direct transcriptional target of ATF3.

**Table 4:** Location of the putative ATF3 binding sequence in the promoter region of the various autophagy-related genes.

Gene Name	Putative ATF3 binding site/s
*Atg101*	−1989
*Atg14*	−1546
*Atg16l2*	−3897, −643, −20
*Atg2b*	473
*Atg4b*	−3943
*Atg4c*	−244
*Atg5*	−22
*Ppp4r1*	−2108

The −5 kb to +1 kb region of the gene containing the putative promoter elements was scanned for the ATF3 binding motif (TGACGTCA) using an in-house written python script. The position of putative ATF3 binding sites relative to the transcription start site in the various genes is listed.

### ATF3 positively regulates JEV replication

**Figure 8:**
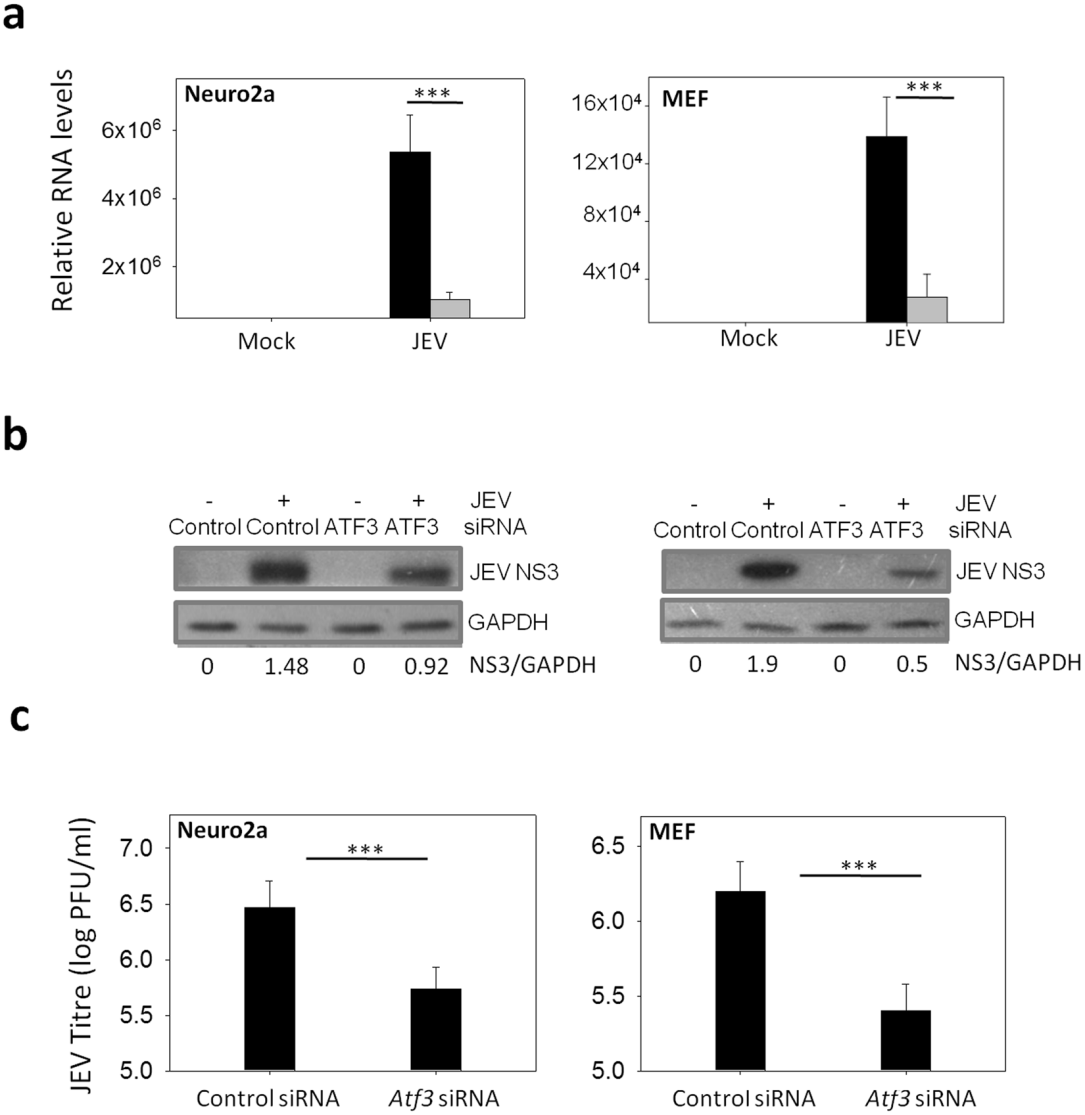
ATF3 depletion leads to reduced JEV replication. (**a**) Neuro2a or MEF cells were treated with either a control non-targeting siRNA or the *Atf3*-specific siRNA for 48 h and then infected with JEV (MOI 5). Total RNA was isolated from the cells at 24 h pi and qRT PCR performed for quantitation of relative levels of JEV RNA. *Gapdh* levels were used for normalization. JEV RNA level in control siRNA-treated cells is shown by the black bar while that in the *Atf3* siRNA-treated cells is shown by the gray bar. (**c**) Cellular supernatant collected from the above experiment was assayed for JEV titer. (**d**) Cell lysates prepared at 24 h pi from Neuro2a cells treated as above were Western blotted for JEV NS3 protein. The ratio of NS3/GAPDH is shown under the figure. The statistical significance of the difference between the data was established by Student’s t-test; *p < 0.05; **p < 0.01; ***p < 0.001.

Loss of ATF3 led to robust induction of antiviral and autophagy pathways, and since both of these have antiviral effects, we investigated the role of ATF3 in JEV replication. To this end, we studied virus replication in ATF3-depleted Neuro2a and MEF cells where a ~60% reduction in JEV RNA was seen in virus-infected cells (Fig. 8a). Concomitantly, the levels of JEV NS1 protein were found to be reduced (Fig. 8b), and the viral yields were significantly suppressed by ~90% in ATF3-depleted Neuro2a and MEF cells (Fig. 8c). These data show that ATF3 is a positive regulator of JEV replication in mammalian cells.

## Discussion

ATF3 is rapidly induced by a range of stress-causing cellular stimuli including ultraviolet radiation, lipopolysaccharides, and cytokines, etc.^24^. Additionally, modulation of ATF3 has been observed in different host cells during diverse virus infections^43–45^. However, the role, if any, of ATF3 in modulating cellular signalling pathways is not well understood. Here, using the data mining and computational biology approach we predicted ATF3 to be a negative regulator of antiviral signalling. These observations were validated by genetic perturbation of ATF3 followed by JEV infection of mammalian cells. Our studies indicate that mammalian cells when infected with JEV, showed a significant induction of ATF3. Interestingly we further observed that loss of ATF3 led to a substantial induction of ISGs even in the absence of type I IFN signalling. Further investigation revealed that ATF3-mediated suppression of antiviral response in the absence of type I IFNs could be due to its ability to directly suppress *Stat1* and *Irf9* genes. Previous studies have shown that ATF3 positively regulates *Stat1* in mouse liver cells by binding to its promoter^46, 47^. In the present work, however, ATF3 was found to negatively regulate *Stat1* in mouse neuronal (Neuro2a) and fibroblast (MEF) cells. This phenomenon may, however, be attributed to the cell-specific role of ATF3, as it has been shown that ATF3 can act as a transcriptional activator or a repressor depending on the cell type and the specific context under which it is induced. For example, ATF3 has been shown to negatively regulate IFN-gamma production in NK cells, whereas it enhanced IFN-gamma production in CD4-positive cells^43, 48^.

Various studies have identified ATF3 induction in response to cytokines, and our observation of ATF3 regulating *Stat1* and *Irf9* suggests that ATF3 could act as a feedback regulator of type 1 IFN responses. Indeed, ATF3 was shown to bind to the *Ifnb1* promoter thereby repressing it in RAW264.7 cells^38^. Since antiviral responses are largely driven by type I IFNs, one can argue that hyper-induction of ISGs following JEV infection in the absence of ATF3 can be attributed to enhanced IFNb1 production. However, in the present study, we show that ATF3 could modulate cellular antiviral and autophagy response in the absence of type I IFN signalling in neuronal cells. Neurons have been reported to be restricted in the production of type I IFNs^39^. Confirming these reports; we also found a lack of IFNb1 secretion in Neuro2a cells post-JEV infection. It was, therefore, intriguing to see robust induction of ISGs in these cells. The induction of antiviral responses despite the deficiency of type 1 IFN responses in Neuro2a cells points towards an unexplored role of ATF3 in regulating antiviral responses. Data presented here show that ATF3 can bind the *Stat1* and *Irf9* promoter. ATF3 could thus regulate the ISGs through direct regulation of ISGF3 complex. Recent reports show that ATF3 regulates the transcription of the gene encoding cholesterol 25-hydroxylase (*Ch25h*), a novel antiviral gene with an important role in virus entry^49^. We also predicted ATF3 binding sites in the promoter region of several ISGs (Table 3) and observed ATF3 binding to *Isg15* promoter using the ChIP assay. It would, therefore, be of interest to further explore the direct regulation of ISGs by ATF3.As negative regulation of inflammatory responses is important to maintain cellular homeostasis, we show in this study that ATF3 can act as a negative regulator of antiviral signaling via multiple nodes.

Apart from the cellular antiviral signalling, various other cellular pathways have been shown to have an antiviral effect. Previously we established the cellular autophagy as a negative regulator of JEV replication^42^. A defective autophagy has been shown to result in perinuclear sequestration of ATF3 leading to increased inflammatory responses in *Atg4b* KO mice, whereas in another report ATF3 was shown to regulate autophagy via Beclin1 pathway, thus suggesting an interplay between autophagy and ATF3^20, 21^. We observed enhanced autophagy in ATF3-depleted cells and found several autophagy-related genes to be highly induced following JEV infection in ATF3-depleted neuronal cells. Further, we found the autophagy-related gene *Atg5* as a transcriptional target of ATF3.There may be additional ATF3 targets among the autophagy-related genes, as many of these had a putative ATF3 binding site but remain to be validated by ChIP assay.

Cellular antiviral and autophagy responses are known to have antiviral effects. Here, we show that ATF3 is a negative regulator of cellular antiviral and autophagy processes. Accordingly, ATF3 depletion led to a significant inhibition of JEV replication in neuronal as well as fibroblast cells. Thus, induction of ATF3, leading to suppression of cellular antiviral and autophagy response, might be exploited by the virus to facilitate its life cycle. Similar findings were made in the case of Lymphocytic Choriomeningitis virus (LCMV) which showed a marked reduction of virus replication in *Atf3* KO BMDMs^29^. Interestingly, however, Coxsackievirus B3 infection of HeLa cells caused suppression of ATF3 expression^45^. Interestingly, Coxsackievirus B3 has been shown to utilize cellular autophagy pathway for its efficient replication suggesting that virus has evolved to inhibit ATF3, thereby inducing autophagy which is beneficial for its replication^50^. On the contrary, ATF3 expression was increased in the livers of mice infected with Murine cytomegalovirus (MCMV) and a striking reduction in viral load was seen in the livers of *Atf3* KO mice relative to WT mice which was attributed to increased IFN-gamma in *Atf3* KO mice^43^.

**Figure 9:**
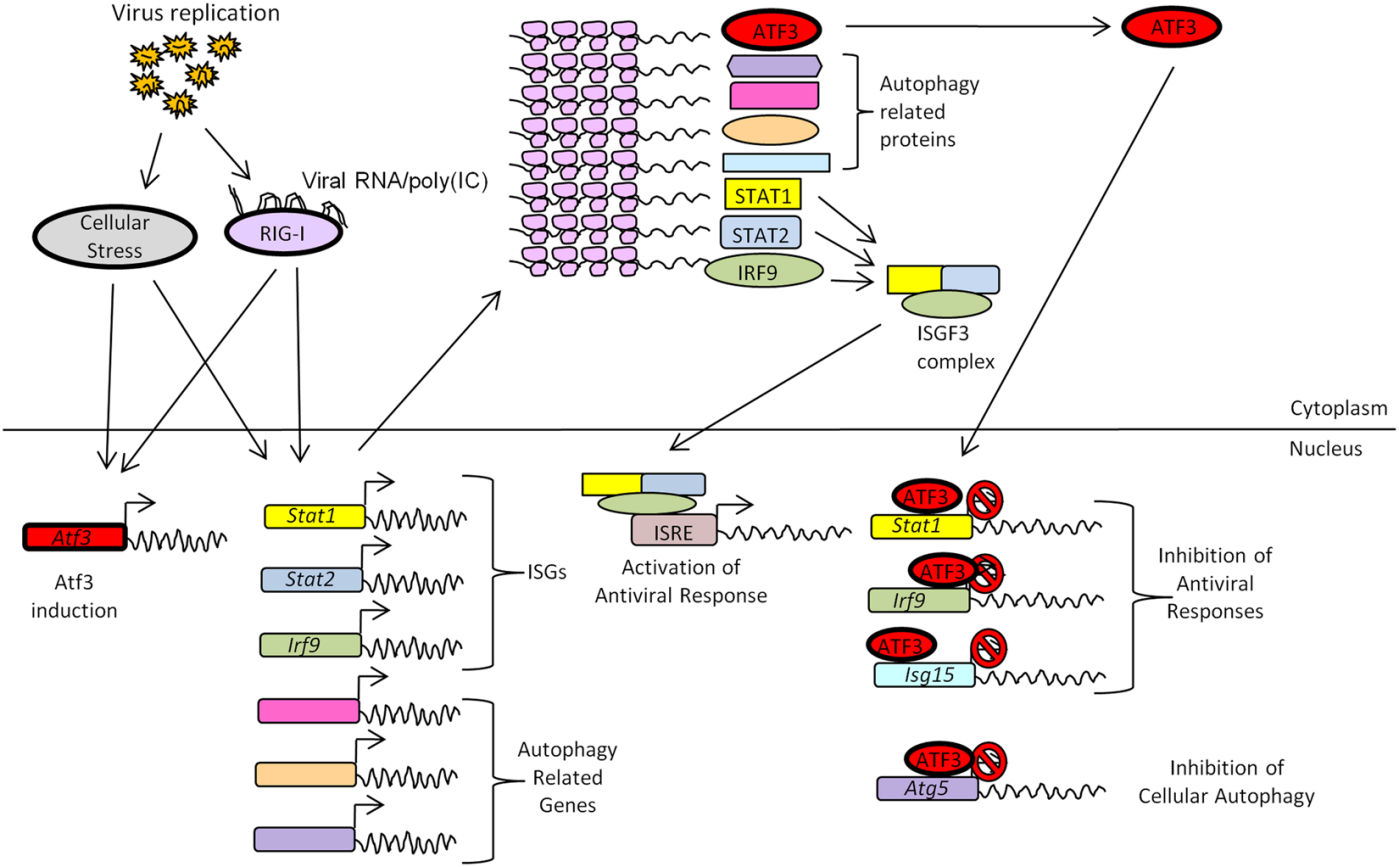
Mechanistic model for ATF3-mediated negative regulation of antiviral and autophagy pathways in the absence of type I IFN. Shown above are the antiviral and autophagy pathways that are triggered following the virus infection of a mammalian cell. Our study shows that ATF3 induced during JEV infection can bind to the promoter region of *Stat1, Irf9*, *Isg15* to suppress the antiviral signaling. We also show ATF3 binding to *Atg5* promoter, thereby suppressing the cellular antiviral pathway. The ATF3, thus, negatively regulates the cellular antiviral signaling and autophagy pathway in the absence of type I IFN.

In summary, we have presented evidence to show ATF3 as a negative regulator of antiviral response and autophagy in mammalian cells (Fig. 9) during JEV infection thereby providing an advantage for the virus to propagate. Importantly, we provide evidence for the type I IFN-independent action of ATF3 in regulating the antiviral genes using the *Stat1-Irf9* axis and the regulation of the cellular autophagy through *Atg5*. This highlights the potential of targeting ATF3 for controlling the JEV replication, although the important role of ATF3 in regulating the cytokine responses cannot be overlooked.
